# Polymorphisms in *TCF7L2* gene are associated with gestational diabetes mellitus in Chinese Han population

**DOI:** 10.1038/srep30686

**Published:** 2016-07-28

**Authors:** Dan Ye, Yang Fei, Qi Ling, Weiwei Xu, Zhe Zhang, Jing Shu, Chengjiang Li, Fengqin Dong

**Affiliations:** 1 Department of Endocrinology and Metabolism, the First Affiliated Hospital, College of Medicine, Zhejiang University, Zhejiang, China; 2 Department of Metabolism and Endocrinology, People’s Hospital of Fuyang City, Zhejiang Province, China; 3 Key Lab of Combined Multi-Organ Transplantation, Ministry of Public Health, Division of Hepatobiliary and Pancreatic Surgery, Department of Surgery, the First Affiliated Hospital, College of Medicine, Zhejiang University, Zhejiang, China; 4 Department of Reproductive Endocrinology, People’s Hospital, Zhejiang Province, China.

## Abstract

This study aimed to investigate the possible association between diabetes susceptibility gene transcription factor 7-like 2 (*TCF7L2*) and gestational diabetes mellitus (GDM) in a Chinese Han population. A total of 556 GDM patients and 500 Non-GDM were included. Eighteen single nucleotide polymorphisms (SNPs) were evaluated. Fifteen tag SNPs were selected from HapMap CHB database with a minor allele frequency of >0.2 and r^2^ of >0.8. Three additional SNPs were also chosen because these SNPs are associated with type 2 diabetes in East Asians. *TCF7L2* rs290487, rs6585194, and rs7094463 polymorphisms were found to be significantly associated with GDM. In multivariate analysis, rs290487 genetic variation (OR = 2.686 per each C allele, *P* = 0.002), pre-BMI > 24 kg/m^2^ (OR = 1.592, *P* = 0.018), age > 25 years (OR = 1.780, *P* = 0.012) and LDL-C > 3.6 mmol/L (OR = 2.034*, P* = 0.009) were identified as independent risk factors of GDM, rs7094463 genetic variation (OR = 0.429 per each G allele, *P* = 0.005) was identified as independent protect factor of GDM. This finding suggests that *TCF7L2* rs290487, and rs7094463 were a potential clinical value for the prediction of GDM.

Gestational diabetes mellitus (GDM) is described as glucose intolerance with onset or first recognition during pregnancy^1^. Based on previous evaluation criteria, GDM affects approximately 5% to 10% of Asian women, with an increasing trend observed in developing countries, including China^2^. GDM can adversely affect maternal and neonatal outcomes. Therefore, health concerns related to GDM have been extensively considered. However, GDM pathogenesis remains unclear. Considering that women with a family history of type 2 diabetes mellitus (T2DM) may be predisposed to an increased risk of GDM^3^ and women with a history of GDM are at an increased risk of developing T2DM later in their lives^4^, we assumed that GDM may share the same risk factors and genetic susceptibilities with T2DM.

Genome-wide association studies (GWAS) have identified more than 90 loci associated with T2DM risk^5^. In a systematic review, GDM risk is significantly associated with nine polymorphisms in seven genes^6^. Several studies have demonstrated the importance of transcription factor 7-like 2 (*TCF7L2*) in regulating glucose metabolism^6^. The T allele of rs7903146 is considered as a risk allele associated with increased *TCF7L2* protein expression and decreased insulin content and secretion^7^,^8^. *TCF7L2*, also known as *TCF4* or the effector of the Wnt signaling pathway, is considered as a master regulator of glucose homeostasis by regulating proinsulin production and processing^7^,^9^. Decreased *TCF7L2* protein levels in T2DM are also correlated with downregulated Gastric Inhibitory Polypeptide (GIP) and glucagon-like peptide-1 (GLP-1) receptors and impaired beta-cell function^10^. In addition, *TCF7L2* affects hepatic glucose metabolism possibly by suppressing gluconeogenesis^11^,^12^,^13^,^14^.

*TCF7L2* are associated with GDM risk in women of different races and ethnicities, however, the relationship between the genetic variants of *TCF7L2* and GDM has not been completely evaluated. This study aimed to evaluate the association between *TCF7L2* and GDM in a Chinese Han population, especially because there had some unique characteristics in Chinese Han diabetic population^15^: Rapid growth of incidence of diabetes mellitus, more higher in post plasma glucose compared to increased fast plasma glucose, rapidly progressive failure of pancreatic islet beta cell in Han population compared to Western populations, growing number of elderly pregnant women, lower body mass index with mild insulin resistance. Our research aims to provide further insights into the mechanisms of genetic variants associated with the risk of GDM.

## Results

### Clinical and biochemical data

The clinical and biochemical parameters of the control and GDM groups are presented in Table 1. The mean age and weeks of gestation of the GDM group were not significantly higher than the Non-GDM group (*P* > 0.05). Pre-BMI, BMI, SBP, DBP, FPG, 1h-PPG, 2h-PPG, Fins, HbA_1c_, TG, TCH, and LDL-C, HOMA-IR and 3h-AUC of insulin were significantly higher in the GDM group than in the Non-GDM group (*P* < 0.001); by contrast, HDL-C, HOMA-B was significantly lower in the GDM group than in the Non-GDM group (*P* < 0.001).

### Genotype distribution and association with GDM

All of the tag SNP MAFs were >20%, MAFs of rs7903146, rs11196205, and rs12255372 was 0.02, 0.03, and 0.01, respectively. All of the 18 SNP frequencies were consistent with Hardy-Weinberg equilibrium (*P* > 0.05, Table 2). Relative strong linkage disequilibrium was found between TCF7L2 rs12255372 and rs11196205 (D′ = 0.675, r^2^ = 0.557), but not others (D′ < 0.6, r^2^ < 0.3) since these SNPs are in high LD with each other that they are not independent of one another.

Among these 18 SNPs, *TCF7L2* rs290487, rs6585194, and rs7094463 were differentially distributed between the GDM and Non-GDM groups (Table 3). In univariate logistic regression analysis, three SNPs were significantly associated with GDM, homozygotes harboring the risk alleles of rs290487 CC genotype yielded 1.661-fold (95% CI = 1.384–1.994, *P* = 5.87E-6) increased risk of GDM, respectively. rs6585194 GG genotype and rs7094463 GG genotype are protecting alleles with 0.683-fold (95% CI = 0.565–0.825, *P* = 5.28E-4) and 0.635-fold (95% CI = 0.472–0.673, *P* = 3.54E-9) decreased risk of GDM, respectively. Therefore, the rs290487 major C-allele, rs6585194 minor C-allele, and rs7094463 minor A-allele were more frequent in the GDM group than in the Non-GDM group. The relation among other SNPs and GDM was not observed (Table 3).

### Characteristics of different SNPs associated with GDM

The C allele of rs290487 showed an association with increased FPG, 2h-PPG, Fins, HbA_1c_ *(P* < 0.05). In rs6585194 and rs7094463, GG genotype carriers showed a lower level of 2h-PPG, Fins and HbA_1c_ than carriers of other genotypes (*P* < 0.001), while the significant difference in FPG was not observed (Table 4).

The rs290487 CC (1.7 vs. 1.2, *P* < 0.001) and TC (1.5 vs. 1.2, *P* < 0.001) genotypes showed significantly higher HOMA-IR indexes than the TT genotype. The CC genotype also presented a higher HOMA-IR than the TC genotype (1.7 vs.1.5, *P* < 0.05). By contrast, HOMA-B level was lower in CC (110.4 vs. 119.2, *P* < 0.01) and TC (117.0 vs. 119.2) than in the TT genotype. Ins 3h-AUC level was also lower in showed in CC and TC than in the TT genotype.

The rs6585194 CG (1.3 vs. 1.5, *P* < 0.05) and GG (1.2 vs. 1.5, *P* < 0.001) genotypes showed significantly lower HOMA-IR indexes than CC genotype; conversely, GG (178.6 vs. 143.4, *P* < 0.001) and GC (146.5 vs. 143.4, *P* < 0.001) genotypes yielded higher Ins 3h-AUC than CC genotype, HOMA-B was also higher in showed in CC and CG than in the GG genotype (*P* = 0.028).

The rs7094463 AG (1.3 vs. 1.5, *P* < 0.05) and GG (1.2 vs. 1.5, *P* < 0.001) genotypes showed significantly lower HOMA-IR indexes than AA genotype; by contrast, GG (181.7 vs. 141.1, *P* < 0.001) and AG (154.3 vs. 141.1, *P* < 0.001) genotypes showed higher AUC than rs7094463 AA genotype, HOMA-B level was higher in GG (116.2 vs. 109.6, *P* < 0.001) and AG (114.7 vs. 199.6) than in the GG genotype. (Table 4).

### Risk factors of GDM: univariate logistic regression analysis and multivariate logistic regression analysis

Clinical parameters that were differentially distributed in different allele of three *TCF7L2* SNPs and rs290487, rs6585194, rs7094463 polymorphisms were considered to be potential influencing factors. The population attributable risk of *TCF7L2* rs290487, rs6585194, rs7094463 were 33.66%, −21.08%, −34.17% respectively. In univariate logistic regression analysis, Pre-BMI, age, systolic pressure, diastolic pressure, LDL-C, *TCF7L2* rs290487, rs6585194 and rs7094463 were showed significant value for GDM and were entered into the multivariate logistic regression analysis. Appropriate cut-off levels were selected for their clinical significance. In Multivariate logistic regression, *TCF7L2* rs290487 genetic variation (OR = 2.686 per each C allele, *P* = 0.002), pre-BMI > 24 kg/m^2^ (OR = 1.592, *P* = 0.018), age > 25 years (OR = 1.780, *P* = 0.012) and LDL-C > 3.6 mmol/L (OR = 2.034, *P* = 0.009) were identified as independent risk factors of GDM, rs7094463 genetic variation (OR = 0.429 per each G allele, *P* = 0.005) was identified as independent protect factor of GDM, while rs7094463 polymorphisms were not been found as independent protect factor of GDM (Table 5).

## Discussion

GDM prevalence increases fast in China, we screened 3,210 Chinese pregnant women and 556 of those participants were diagnosed with GDM, GDM incidences has increased by 17.3% compared with previously reported data of epidemiological surveys (5% to 10%). This increase can be attributed to dependence on novel diagnostic criteria^16^, which recommend lower diagnostic cut-point than previous criteria. Thus far, GDM pathogenesis has not been completely elucidated.

In addition to clinical implications: Higher BP, higher level of TG, LDL-C, Ins 3-hAUC, HOMA-IR and lower HOMA-B in GDM group, genetic factors are likely involved in GDM development. The *TCF7L2* gene has been regarded as the most common susceptible gene for T2DM among various ethnic groups in the world. Genome-wide association studies have identified several potent diabetes susceptibility loc^5^,^17^,^18^, which have been further identified among various populations, including Chinese Han population^19^,^20^,^21^. However, genetic backgrounds, including risk allele frequency and linkage disequilibrium distribution of SNPs, differ between East Asians and Caucasians^22^,^23^. Our study found that *TCF7L2* rs290487, rs6585194, and rs7094463 polymorphisms were significantly associated with GDM. The rs290487 major C-allele, rs6585194 minor C-allele, and rs7094463 minor A-allele showed increased FPG, 2h-PPG, Fins, HbA_1c_. *TCF7L2* rs7903146 risk variants are associated with T2DM in European-derived populations; however, this relationship has not been found in our study, same to several East Asians study^18^,^21^,^23^,^24^,^25^,^26^. These inconsistent findings may be attributed to the low risk allele frequency of rs7903146, which is approximately 0.02 in East Asians^20^,^22^. Therefore, SNPs with high MAF (>0.20) were selected to further elucidate the correlation between diabetes susceptibility genes and GDM. *TCF7L2* rs290487 contains a more common genetic variant (MAF: 0.35 *vs.* 0.02) and exhibits greater significance than rs7903146 in Chinese Han population^20^,^22^. The results were further duplicated by other Chinese studies^27^,^28^.

Our results demonstrated that *TCF7L2* rs290487, rs6585194 and rs7094463 polymorphisms were correlated with insulin resistance and insulin secretion of patients with GDM. Patients with rs290487 major C-allele, rs6585194 minor C-allele, and rs7094463 minor A-allele were showed a significantly higher HOMA-IR, lower HOMA-B and Ins 3h-AUC. This result was consistent with a previous study, which revealed that rs290487 C allele is significantly associated with increased insulin resistance among Taiwanese and Caucasians^26^. HOMA-B and Ins 3-hAUC were lower in rs290487 CC homozygote than in other genotypes. Although genetic variants in *TCF7L2* gene likely increase the risk of diabetes, the variant allele of *TCF7L2* rs6585194 and rs7094463 elicited protective effects against diabetes. The G-allele of both SNPs significantly decreased insulin resistance and increased insulin secretion. Therefore, the variant allele of *TCF7L2* rs6585194 and rs7094463 decreased GDM probability. This result indicated that *TCF7L2* rs290487 rs6585194 and rs7094463 polymorphisms were also correlated with insulin secretion among patients with GDM. The association of *TCF7L2* genetic variant with increased insulin resistance and decreased insulin secretion may help understand GDM pathogenesis. *TCF7L2* gene polymorphisms are also associated with increased hepatic glucose production and reduced hepatic insulin sensitivity and regulated the hepatic glucose metabolism via the gluconeogenesis pathway in humans.

Multivariate logistic regression analysis showed that *TCF7L2* rs290487 CC genotype, Pre-BMI, LDL-C level and age were independent risk factors of GDM which were confirmed in several studies^29^,^30^. *TCF7L2* rs7094463 is independent protective factor of GDM, while *TCF7L2* rs6585194 GG genotype were associated with GDM but were not independent risk factors. *TCF7L2* also is an important transcription factor for the execution of downstream signals of the Wnt/b-catenin/*TCF* pathway, which has been shown to regulate hepatic glucose metabolism through the modulation of multiple pathways, such as the insulin signal transduction pathway (insulin receptor substrate-1/Phosphatidyl Inositol 3-kinase: IRS-1/PI3K).

Several limitations of this study were found. Although this study included 556 women with GDM and 500 control subjects, the statistical power of the sample was not sufficiently large to detect a weak effect size (*OR* < 1.2). As a result, other associations may have been overlooked. Furthermore, this study failed to determine whether all of the subjects in the control group experienced pregnancy without GDM.

In summary, the data suggested that the genetic variation rs290487 and rs7094463 in the *TCF7L2* gene were independent influencing factors of GDM in the Chinese Han population, *TCF7L2* gene might to be one of the candidate genes for confering susceptibility to gestational diabetes in Chinese Han people.

## Materials and Methods

### Patient characteristics

Pregnant Chinese Han women with or without GDM at our institution were evaluated. Written informed consent was obtained from each subject who participated in the study. This study was approved by the Medical Ethical Committee of the First Affiliated Hospital, College of Medicine, Zhejiang University. The methods were carried out in accordance with the approved guidelines.

A total of 3,210 Chinese pregnant women were screened for GDM in three local hospitals in Hangzhou from January 2014 to December 2014. Pregnant women without a previous diagnosis of glucose intolerance or diabetic family history were routinely screened for GDM between 24 and 28 weeks of gestation. These pregnant women were subjected to a 75 g oral glucose tolerance test (OGTT). GDM diagnosis was based on the criteria set by the American Diabetes Association^16^. Glucose threshold values were listed as follows: 5.1 mmol/L during fasting, 10.0 mmol/L for 1 h, and 8.5 mmol/L for 2 h. GDM was diagnosed if one or more of glucose concentrations satisfied or exceeded the threshold value. Based on these criteria, 556 of 3,210 participants were diagnosed with GDM and 2,654 were diagnosed with normal glucose tolerance (NGT). A total of 556 patients with GDM and 500 age-matched participants with NGT were recruited as controls(Non-GDM), and 34 of GDM whose fasting plasma glucose (FPG) exceeded 7.0 mmol/L or two-hour post-challenge plasma glucose (2h-PPG) exceeded 10 mmol/L were received the insulin treatment of Novolin 30R. The average dose of insulin used in prepartal was 28u/D (0.48 u/Kg).

Clinical and biochemical data of the subjects were collected at 24 weeks to 28 weeks of gestation. Clinical data included age, height, weight, weight before pregnancy (consulting the initial medical record of pregnancy registry), systolic blood pressure, and diastolic blood pressure (GDM were measured once a week and meanwhile were required to measure everyday by themselves, Non-GDM were measured thrice at the screening day). A family history of T2DM in each subject was also recorded. The body mass index of mothers before pregnancy (pre-BMI) and BMI during the test were calculated. Biochemical data consisted of FPG, 1hPPG, 2hPPG, fasting plasma insulin (FIns), glycated hemoglobin (HbA_1c_), serum triacylglycerol (TG), total cholesterol (TCH), high-density lipoprotein cholesterol (HDL-C), and low-density lipoprotein cholesterol (LDL-C). FPG, 1hPPG, 2hPPG, TCH, TG, HDL-C, LDL-C concentrations were determined by commercial enzymatic methods (test kits from Shanghai Rongsheng Biotech, Inc, Shanghai, China). Fins was determined by chemiluminometry (SIEMENS) and HbA_1c_ was determined by high-pressure liquid chromatography. Homeostatic model assessment (HOMA) data and area under the curve (AUC) of insulin in 75 g OGTT performed at the time of GDM diagnosis were calculated to assess insulin resistance and beta-cell function. Homeostasis model assessment of insulin resistance (HOMA-IR) was determined using the following equation: (FIns in mU/L × FPG in mmol/L)/ 22.5. Homeostasis model assessment of beta-cell function (HOMA-B) was also quantified using the following equation: (FIns in mU/L × 20)/ (FPG in mmol/L-3.5)^31^. The AUC of insulin at 3 h, another assessment index of beta-cell function, was evaluated according to trapezoid method: *V*_1_ + *V*_2_ + 0.5 × *V*_0_ + 0.5 × *V*_3_, where *V* is the insulin concentration at the indicated time^32^.

### Genotyping

Genomic DNA was isolated from ethylenediaminetetraacetic acid-anticoagulated whole blood of recipients by using a QIAamp DNA blood mini kit (Qiagen, Hilden, Germany). Single nucleotide polymorphisms (SNPs) in the most significant diabetes-susceptibility locus *TCF7L2* were selected from HapMap CHB database^17^ (public data release 21a/phaseII, January 2007; http://snp.cshl.org/cgi-perl/gbrowse/ hapmap22_B36/) with a minor allele frequency (MAF) of >0.2 and *r*^*2*^ of >0.8. Fifteen tag SNPs in *TCF7L2* (rs10749127, rs10787475, rs11196224, rs12775879, rs17130188, rs290481, rs290487, rs290489, rs3750804, rs4918792, rs6585194, rs7085532, rs7094463, rs7919409, and rs966227) were chosen and analyzed. Three additional SNPs in *TCF7L2* gene (rs7903146, rs11196205, and rs12255372) were also selected because these SNPs are also significantly associated with T2DM among East Asians^22^,^33^,^34^. Allele frequencies were all in Hardy-Weinberg equilibrium (*P* > 0.05, Table 2). All primers were designed using Primer3 software (http://frodo.wi.mit.edu/primer3/).Applied Biosystems SNaPshot and TaqMan technology were applied to identify genetic polymorphism. The fragment was first amplified by polymerase chain reaction (PCR). Each PCR reaction contained 1× HotStar Taq buffer, 3.0 mM Mg^2+^, 0.3 mM dNTP, 0.1 mM of each primer, 1U HotstartTaq polymerase (Qiagen Inc., Hilden, Germany). The second step was the multiple single-base reaction, which included 5 uL SNaPshot Multiplex Kit (Applied Biosystems, Foster City, California, USA). The detailed procedure was described in a previous study^35^.

### Statistical Analysis

Quantitative variable was expressed as mean ± standard deviation or interquartile range (25–75%) and the continuous data (HOMA-B, HOMA-IR, and AUC of insulin) were log-transformed to approximate normal distributions. Quantitative variables were compared using Student’s *t*-test or Mann-Whitney test; categorical variables were compared using chi-square test. Linkage between SNPs was analyzed using pairwise linkage disequilibrium methods evaluating *r*^*2*^ and *D′*. Correlations were evaluated through Pearson linear regression. Risk factors were evaluated through logistic regression analysis. Variables with statistical significance in univariate analysis were subjected to stepwise multivariate logistic regression analysis. Hardy-Weinberg equilibrium, linkage disequilibrium, and haptotype were analyzed using Haploview software and SNP Stats web tool (http://bioinfo.Iconcologia.net/snpstats/start.htm). Genotypes were assigned with the following codes: 0, 1, and 2 copies of the minor allele; odds ratio (*OR*) was expressed per difference in the number of risk alleles. An univariate logistic regression analysis model for all 18 SNPs and other clinical parameter (including Pre-BMI, age, family history of diabetes, gravidity, systolic pressure, diastolic pressure, TG, TCH, LDL-C, HDL-C) was performed, multiple logistic regression model (including Pre-BMI, age, systolic pressure, diastolic pressure, LDL-C, and *TCF7L2* rs290487, rs6585194 and rs7094463) was used to investigate the individual association of these SNPs on GDM. These analyses were based on additive, recessive, and dominant models and adjusted for age and family history of T2DM. *ORs* with 95% confidence intervals (*CIs*) were presented. Multiple linear regression models adjusted for age were also applied to analyze quantitative traits. Other analyses were performed using *SPSS* version 11.0 (*SPSS* Inc., Chicago, IL). Benjamini-Hochberg method was used to control the false discovery rate (FDR) in the unconditional logistic regression analysis^36^. A two-sided *P* value of <5.00E-04 was considered statistically significant. The population attributable risk (PAR: Ie - Iu (Ie = Incidence in exposed, Iu = incidence in unexposed).

## Additional Information

**How to cite this article**: Ye, D. *et al*. Polymorphisms in *TCF7L2* gene are associated with gestational diabetes mellitus in Chinese Han population. *Sci. Rep.* **6**, 30686; doi: 10.1038/srep30686 (2016).

## Figures and Tables

**Table 1:** Anthropometric and laboratory data of the studied groups.

	non-GDM (n = 500)	GDM (n = 556)	*p* value
Age (years)	28.9 ± 5.8	29.2 ± 4.5	0.334
Pre-BMI (kg/m^2^)	21.4 ± 2.1	22.4 ± 2.6	<0.001
BMI (kg/m^2^)	26.7 ± 2.7	27.8 ± 2.3	<0.001
Weeks of gestation	26.3 ± 1.0	26.5 ± 1.8	0.620
SBP (mmHg)	110.0 ± 5.5	115.4 ± 6.7	<0.001
DBP (mmHg)	68.5 ± 3.4	70.8 ± 3.6	<0.001
FPG (mmol/l)	4.5 ± 1.6	4.7 ± 2.0	<0.001
1-h 75 g glucose (mmol/l)	7.5 ± 1.8	12.8 ± 3.3	<0.001
2-h 75 g glucose (mmol/L)	6.4 ± 1.9	8.9 ± 2.1	<0.001
HbA_1c_ (%)	5.1 ± 1.2	5.6 ± 1.3	<0.001
TC (mmol/l)	6.1 ± 1.0	6.2 ± 1.0	<0.001
HDL-C (mmol/l)*	2.2 ± 0.6 (1.8, 4.5)	2.1 ± 0.6 (1. 5, 3.8)	<0.001
LDL-C (mmol/l)*	3.1 ± 0.7 (2.4, 5.6)	3.3 ± 0.8 (2.9, 6.8)	<0.001
TG (mmol/l)*	2.1 ± 0.8 (1.1, 4.8)	2.5 ± 0.90 (1.6, 3.6)	<0.001
FIns (mu/l)	6.0 ± 1.7	7.4 ± 1.7	<0.001
Ins 3-h AUC (mu·l^−1^·h)	146.7 ± 21.4	191.2 ± 26.1	<0.001
HOMA-IR	1.3 ± 0.2	1.5 ± 0.2	<0.001
HOMA-B	119.1 ± 14.1	113.0 ± 12.3	<0.001

Non-GDM, non gestational diabetes mellitus; GDM, gestational diabetes mellitus; Pre-BMI, body mass index of mothers before pregnancy; BMI, body mass index of measurement during the test; SBP, systemic blood pressure; DBP, diastolic blood pressure; FPG, fasting plasma glucose; 1-h 75 g glucose, one-hour post- challenge plasma glucose; 2-h 75 g glucose, two-hour post-challenge plasma glucose; TC, total cholesterol; HDL-C, high-density lipoprotein cholesterol; LDL-C, low-density lipoprotein cholesterol; TG, serum triacylglycerol; Fins, fasting plasma insulin; Ins 3-h AUC, total of plasma insulin area under the curve in three hours; HOMA-IR, homeostasis model assessment of insulin resistance; HOMA-B, homeostasis model assessment of beta-cell function.

^*^M-W test.

**Table 2:** Allele frequency and Hardy-Weinberg equilibrium of SNPs in *TCF7L2*.

SNP				HapMap		This study		HWE P
				Genotype(%)			Major/minor Allele		MAF	Minor Allele	MAF
rs10749127	CC (0.374)	CT (0.475)	TT (0.151)	C/T	0.389	T	0.30	0.62
rs10787475	TT (0.588)	TC (0.357)	CC (0.054)	T/C	0.233	C	0.27	0.61
rs11196224	CC (0.347)	CT (0.484)	TT (0.168)	C/T	0.411	T	0.31	0.70
rs12775879	TT (0.623)	TG (0.333)	GG (0.045)	T/G	0.211	G	0.25	0.68
rs17130188	TT (0.460)	TC (0.437)	CC (0.104)	T/C	0.322	C	0.43	0.20
rs290481	AA (0.430)	AG (0.451)	GG (0.118)	A/G	0.344	G	0.35	0.94
rs290487	TT (0.412)	TC (0.456)	CC (0.124)	T/C	0.352	C	0.28	0.85
rs290489	GG (0.412)	GA (0.456)	AA (0.124)	G/A	0.352	A	0.34	0.94
rs3750804	CC (0.387)	CT (0.470)	TT (0.143)	C/T	0.378	T	0.23	0.79
rs4918792	GG (0.347)	GA (0.484)	AA (0.169)	G/A	0.411	A	0.48	0.90
rs6585194	CC (0.334)	CG (0.488)	GG (0.178)	C/G	0.422	G	0.33	0.74
rs7085532	AA (0.334)	AG (0.488)	GG (0.178)	A/G	0.422	A	0.40	0.58
rs7094463	AA (0.262)	AG (0.500)	GG (0.238)	A/G	0.488	G	0.44	0.83
rs7919409	TT (0.554)	TC (0.380)	CC (0.066)	T/C	0.256	C	0.35	0.10
rs966227	CC (0.250)	CT (0.500)	TT (0.250)	C/T	0.500	T	0.34	0.80
rs7903146	CC (0.956)	CT (0.043)	TT (0.001)	C/T	0.022	T	0.02	0.93
rs11196205	GG (0.935)	GC (0.064)	CC (0.001)	G/C	0.033	C	0.03	0.95
rs12255372	GG (0.978)	GT (0.022)	TT (0.000)	G/T	0.011	T	0.01	0.89

MAF, Minor allele frequency; HWE, Hardy-Weinberg equilibrium; SNP, single-nucleotide polymorphism.

**Table 3:** Genotype, allele frequencies and logistic regression analysis of polymorphisms with significant association with GDM.

logistic regression, n (%)							
	Genotype	GDM	non-GDM	OR	95% CI	*p*	*p*-adjusted
rs290487	TT	223 (40)	240 (48)	1			
(T/C)	TC	223 (40)	235 (47)	1.021	(0.789, 1.323)	0.873	
	CC	110 (20)	25 (5)	4.735	(2.957, 7.584)	8.35E-10	
	T allele	669 (60)	715 (72)	1			
	C allele	443 (40)	285 (28)	1.661	(1.384, 1.994)	5.87E-6	5.28E-5
rs6585194	CC	334 (60)	222 (44)	1			
(C/G)	CG	165 (30)	227 (47)	0.483	(0.372, 0.628)	2.75E-7	
	GG	57 (10)	51 (9)	0.743	(0.491, 1.124)	0.159	
	C allele	833 (75)	671 (67)	1			
	G allele	279 (25)	329 (33)	0.683	(0.565, 0.825)	5.28E-4	3.15E-3
rs7094463	AA	267 (48)	152(30)	1			
(A/G)	AG	232 (42)	251 (50)	0.526	(0.403, 0.688)	1.74E-6	
	GG	57 (10)	97 (20)	0.335	(0.228, 0.491)	4.12E-8	
	A allele	766 (69)	555 (56)	1			
	G allele	346 (31)	445 (44)	0.635	(0.472, 0.673)	3.54E-9	6.32E-8

GDM, gestational diabetes mellitus; Non-GDM, non gestational diabetes mellitus; OR, odds ratio; 95% CI, 95% confidence intervals. p-adjusted value was calculated using B-H method^36^.

**Table 4:** Patient characteristics of different SNPs associated with GDM.

N	TT	TC	CC	*p* value TT vs. CC
	223	223	110	
rs290487				
FPG (mmol/l)	4.5 ± 1.4	4.7 ± 1.6	4.8 ± 1.7	0.022
2hPPG (mmol/l)	7.1 ± 2.0	8.0 ± 1.9	8.6 ± 1.9	7.13E-15
FIns (mu/l)	6.1 ± 1.6	7.0 ± 1.6	7.2 ± 1.7	8.33E-13
HbA_1c_ (%)	5.2 ± 1.1	5.5 ± 1.2	5.7 ± 1.2	4.06E-06
HOMA-IR	1.2 ± 0.2	1.5 ± 0.2	1.7 ± 0.2	1.99E-104
HOMA-B	119.2 ± 14.1	117.0 ± 13.4	110.4 ± 13.0	4.26E-11
Ins 3-h AUC (mu·l^−1^·h)	190.1 ± 23.5	163.2 ± 26.3	155.6 ± 21.4	6.28E-57
N	CC	CG	GG	*p* value CC vs. GG
	334	165	57	
rs6585194				
FPG (mmol/l)	4.7 ± 1.7	4.6 ± 1.7	4.5 ± 1.9	0.125
2hPPG (mmol/l)	8.4 ± 1.9	7.0 ± 1.7	6.5 ± 1.8	3.28E-21
FIns (mu/l)	7.3 ± 1.7	6.1 ± 1.7	5.9 ± 1.7	2.27E-14
HbA_1c_ (%)	5.5 ± 1.0	5.2 ± 1.2	5.1 ± 1.1	9.17E-05
HOMA-IR	1.5 ± 0.2	1.3 ± 0.2	1.2 ± 0.2	8.72E-56
HOMA-B	121.0 ± 13.9	119.5 ± 14.2	118.2 ± 13.8	0.028
Ins 3-h AUC (mu·l^−1^·h)	143.4 ± 19.2	146.5 ± 21.9	178.6 ± 22.0	1.62E-65
N	AA	AG	GG	*p* value AA vs. GG
	267	232	57	
rs7094463				
FPG (mmol/l)	4.8 ± 1.8	4.6 ± 1.7	4.6 ± 1.8	0.223
2hPPG (mmol/l)	8.4 ± 1.8	7.5 ± 1.8	6.5 ± 1.8	2.33E-13
FIns (mu/l)	7.3 ± 1.7	6.4 ± 1.8	6.0 ± 1.7	3.99E-29
HbA_1c_ (%)	5.7 ± 1.2	5.2 ± 1.0	5.1 ± 1.1	2.93E-08
HOMA-IR	1.5 ± 0.2	1.3 ± 0.2	1.2 ± 0.2	6.81E-53
HOMA-B	109.6 ± 12.9	114.7 ± 13.2	116.2 ± 14.0	3.67E-07
Ins 3-h AUC (mu·l^−1^·h)	141.1 ± 18.7	154.3 ± 20.8	181.7 ± 21.5	1.48E-112

Pre-BMI, body mass index of mothers before pregnancy; FPG, fasting plasma glucose; 2-h 75 g glucose, two -hour post-challenge plasma glucose; TC, total cholesterol; LDL-C, low-density lipoprotein cholesterol; Fins, fasting plasma insulin; HOMA-IR, Homeostasis model assessment of insulin resistance; HOMA-B, Homeostasis model assessment of beta-cell function; Ins 3-h AUC, total of plasma insulin area under the curve in three hours.

**Table 5:** Univariate and multivariate logistic regression analysis of risk factors associated with GDM.

Factor	Univariate		Multivariate	
	*p* value	Odds ratio (95% CI)	*p* value	Odds ratio (95% CI)
Age >25 years (0 = no, 1 = yes)	0.049	1.289 (1.006, 1.652)	0.012	1.780 (1.391, 2.277)
Pre-BMI > 24 kg/m^2^ (0 = no, 1 = yes)	0.003	1.456 (1.138, 1.865)	0.018	1.592 (1.248, 2.531)
LDL-C level > 3.6 mmol/l (0 = no, 1 = yes)	<0.001	1.780 (1.391, 2.277)	0.009	2.034 (1.561, 3.239)
SBP > 140 mmHg (0 = no, 1 = yes)	0.074	1.884 (0.980, 3.469)		
DBP > 90 mmHg (0 = no, 1 = yes)	0.162	1.527 (0.840, 2.775)		
rs290487 genotype (0 = TT, 1 = TC, 2 = CC)	0.001	4.735(2.957, 7.584)		2.686 (1.979, 4.372)
rs6585194 genotype (0 = CC, 1 = CC, 2 = GG)	0.027	0.628(0.4141, 0.951)		
rs7094463 genotype (0 = AA, 1 = GA, 2 = GG)	<0.001	0.335(0.228, 0.491)	0.005	0.429 (0.329, 0.559)

Pre-BMI, body mass index of mothers before pregnancy; LDL-C, low-density lipoprotein cholesterol; SBP, systemic blood pressure; DBP, diastolic blood pressure; 95% CI, 95% confidence interval.
